# Investigation of In-Vitro Antioxidant and Electrochemical Activities of Isolated Compounds from *Salvia chamelaeagnea* P.J.Bergius Extract

**DOI:** 10.3390/antiox8040098

**Published:** 2019-04-12

**Authors:** Ninon G.E.R. Etsassala, Adewale O. Adeloye, Ali El-Halawany, Ahmed A. Hussein, Emmanuel I. Iwuoha

**Affiliations:** 1 Chemistry Department, University of the Western Cape, Private Bag X17, Bellville 7535, South Africa; 3415216@myuwc.ac.za (N.G.E.R.E.); eiwuoha@uwc.ac.za (E.I.I.); 2 Chemistry Department, Cape Peninsula University of Technology, Symphony Rd., Bellville 7535, South Africa; aowale@gmail.com; 3 Pharmacognosy Department, Faculty of Pharmacy, Cairo University, Cairo 11562, Egypt; ahalawany2003@hotmail.com

**Keywords:** *Salvia chamelaeagnea*, antioxidant activity, cyclic voltammetry, oxidative stress, anti-ageing properties, abietane diterpenes

## Abstract

We have investigated the in-vitro antioxidant activity and electrochemical redox properties of a number of natural compounds (carnosol, carnosic acid, 7-ethoxyrosmanol, ursolic acid, rosmanol and ladanein) isolated from the methanolic extract of *Salvia chamelaeagnea* collected from the Cape floristic region, South Africa. The results from trolox equivalent antioxidant capacity (TEAC), ferric-ion reducing antioxidant parameter (FRAP) oxygen radical absorbance capacity (ORAC), as well as the inhibition of Fe^2+^-induced lipid peroxidation showed strong antioxidant capacities for carnosol and rosmanol. A structural analysis of the compounds suggests that multiple OH substitution, conjugation and lactone ring in carnosol and rosmanol are important determinants of the free radical scavenging activity and electrochemical behavior. Pharmacophore generated demonstrates H-donor/acceptor capabilities of the most active compounds. Rosmanol, when compared to other compounds, exhibits the lowest oxidation potential value with an anodic peak potential (E_pa_) value of 0.11 V, indicating that rosmanol has the highest antioxidant power, which is in good agreement with ORAC and lipid peroxidation experiments. The lipophilic nature of carnosol, carnosic acid and rosmanol enhanced their absorption and activity against oxidative stress related to the treatment of age-related diseases. These results confirm the first report on the in-vitro antioxidant and electrochemical activities of *S. chamelaeagnea* constituents and underline the medicinal uses of this plant as natural preservatives for skin ageing or in pharmaceutical applications.

## 1. Introduction

Natural products and plant materials have been used since time immemorial in diverse applications including cosmetics. In recent times, they have become increasingly useful in the prevention of skin hyper-pigmentation through the inhibitory action of tyrosinase enzyme [1]. A set of structurally related phenolic compounds have been found to play an important role in the neutralization of free radicals [2], reducing oxidative stress and other related diseases associated with the over-accumulation of free radicals such as brain disorder, diabetes, cancer, heart disease and immune response dismiss [3]. Numerous factors behind the accumulation of reactive oxygen species (ROS) in the body have been previously highlighted; these include: continuous contact with series of environmental pollutants, lifestyle (smoking, alcohol consumption), when above threshold level, can cause oxidative stress by damaging important intracellular macromolecules such as protein, lipids and deoxyribonucleic acid (DNA) [4].

*Salvia chamelaeagnea* belongs to the genus *Salvia* (Lamiaceae), with about 900 species worldwide, and only 26 among them are indigenous to Southern Africa. It is a flowering plant commonly known as “sage” which originates from the South Western part of the Cape of Good Hope [5]. Literature shows that *S. chamelaeagnea* has been used for medicinal purposes in the treatment of coughs, colds, and heartburn [6]. It has also been documented that the extract of this plant exhibits pharmaceutical activities including antioxidant [3], anti-microbial and bacterial infections [6,7]. *Salvia* species have been reported to be rich source of phytochemicals constituents including phenolic acid, polyphenols and flavonoids. Among these phytochemical constituents are carnosol and carnosic acid which are the most abundant that contribute about 90% of the antioxidant activity of Lamiaceae family [8]. Previous chemical and biological studies also showed some terpenoids like carnosol, carnosic acid, ursolic acid and its isomer oleanolic acid with notable antibacterial activities against *Staphylococcus aureus* [5].

This work primarily examines the antioxidant, as well as the anti-tyrosinase activities of six compounds isolated from *S. chamelaeagnea*. Their electrochemical properties and structure-activity relationships were used to explain the observed effects and suggest the mechanism of reaction.

## 2. Experimental Section

### 2.1. Plant Collection

The fresh leaves of *Salvia chamelaeagnea* P.J.Bergius was identified by Christopher Cupido (South African National Biodiversity Institute (SANBI), Kirstenbosch, Cape Town, South Africa) and collected from Cape Flats Nature Reserve, Western Cape, South Africa (Supplementary Materials). A voucher specimen with herbarium number NBG1465544-0 was deposited at the Compton Herbarium, Kirstenbosch.

### 2.2. Chemical and Reagents

Nuclear magnetic resonance (^1^H- and ^13^C-NMR) spectra were recorded on Avance 400 MHz NMR spectrometer (Bruker, Rheinstetten, Germany) in CDCl_3_ using tetramethylsilane (TMS) as the internal reference standard. Preparative high-performance liquid chromatography (HPLC) column separation technique using HPLC grade Methanol and de-ionized water was further used in the purification of isolated compounds.

### 2.3. Extraction and Purification of Chemical Constituents

The leaves of fresh plant material (1.09 kg) were grounded and extracted with methanol (4.50 L) at room temperature (25 °C) for 24 h. The methanolic extract was filtered and evaporated to dryness under reduced pressure using a rotary evaporator working at 40 °C. Of the 29.55 g total crude extract obtained (2.71% yield), 29 g was applied to a silica gel column (30 × 18 cm) and eluted using gradient of hexane (Hex) and ethyl acetate (EtOAc) in order of increasing polarity: Sixty-four (64) fractions (500 mL each) were collected and combined according to their thin layer chromatography profiles resulting in fifteen fractions, which were labeled I to XV. Fraction VII (0.55 g) was subjected to re-chromatography column on Sephadex LH-20 and then eluted with aqueous-ethanol (5:95), to afford carnosol as compound 1 (0.19 g, 0.017%). Fraction VIII (0.50 g) was further re-chromatographed on Sephadex LH-20 using same solvent system of aqueous-ethanol (5:95) to isolate carnosic acid as compound 2 (0.09 g; 0.0082%). Sub-fraction VIII-6 (50 mg) obtained from fraction VIII, was injected to the HPLC and eluted with gradient solvent system of MeOH and de-ionized water (3:1 to 100% MeOH in 40 min) to isolate 7-ethoxyrosmanol (**3**, 28 min, 0.037 g; 0.0034%). Other sub-fractions (VIII-2 and VIII-5) from fraction VIII, gave ursolic acid (**4**, R_t_ 22.1 min, 0.014 g; 0.0012%), and rosmanol (**5**, R_t_ 32.1 min, 0.007 g; 0.0000064%) via the same HPLC procedure.

Compound 6 (ladanein (**6**, R_t_ 12.7 min, 0.136 g, 0.0037%) was isolated from fraction XIV (0.50 g) on Sephadex LH-20 column using aqueous-methanol (1:9, *v*/*v*) which was further purified on HPLC gradient elution for 45 min in MeOH and de-ionized water (3:1, *v*/*v*).

### 2.4. Anti-Tyrosinase Assay

This assay was performed using the method previously described with slight modifications [9]. Samples were dissolved in DMSO (dimethyl sulphoxide) to a stock solution of 1 mg/mL (*w*/*v*). Further dilutions were done with phosphate buffer (pH 6.5) for all working solutions to the concentrations of 1000, 500, 100, 50, 10 μg/mL. Kojic acid was used as a positive control. In each well of a 96-well plate, 70 μL of each sample working solution (extract/pure compounds) was combined with 30 μL of tyrosinase [from mushroom, 500 Units/mL in phosphate buffer (pH 6.5)] in triplicate. After incubation at room temperature for 5 min, 110 μL of substrate (2 mM L-tyrosine) was added to each well. The sample control was made up of each sample with phosphate buffer in the absence of tyrosinase enzyme. The reacting mixture was then incubated for 30 min at room temperature. The enzyme activity was determined by measuring the absorbance at 490 nm using plate reader. The percentage of tyrosinase inhibition was calculated as follows.

[(A − B) − (C − D)]/(A − B) × 100(1)

where A is the absorbance of the control with the enzyme, B is the absorbance of the control without the enzyme, C is the absorbance of the test sample with the enzyme and D is the absorbance of the test sample without the enzyme.

### 2.5. Anti-Oxidant Assays

#### 2.5.1. Ferric-Ion Reducing Antioxidant Power Assay (FRAP)

The FRAP assay was carried out in accordance to the method described previously [10].

#### 2.5.2. Trolox Equivalent Absorbance Capacity (TEAC) Assay

The total antioxidant activity of the test samples was measured by the method as described previously [11].

#### 2.5.3. Oxygen Radicals Absorbance Capacity (ORAC) Assay

ORAC assay was done according to the previous method with slight modifications [12]. 

#### 2.5.4. Inhibition of Fe (II)-Induced Microsomal Lipid Peroxidation Assay

The lipid peroxidation assay was carried out by the method as described previously [13], with a few modifications adopted. All experiments were performed in triplicates at room temperature. The reaction mixture contained microsomes (1 mg of protein/mL in 0.01 M potassium phosphate buffer; pH 7.4, supplemented with 1.15% KCl). The positive control includes microsomes, buffer and ferrous sulphate, in the absence of the samples to be tested. The sample stock solutions *Salvia chamelaeagnea* (SC) and **1**–**6**) were prepared in DMSO (1 mg/mL, *w*/*v*). The working sample solutions were prepared in 0.01 M potassium phosphate buffer pH 7.4, supplemented with 1.15% KCl diluted to 100, 50 and 10 μg/mL concentrations. 100 μL of each sample (working solutions) were dissolved in potassium phosphate buffer and pre-incubated with 500 μL microsomes a 37 °C for 30 min in a shaking water bath. 200 μL of KCl-buffer were added to the mixture, followed by 200 μL of a 2.5 mM ferrous sulfate solution and incubated at 37 °C for 1 h in a shaking water bath. The reaction was terminated with 10% trichloroacetic acid (TCA) solution (1 mL) containing 125 μL butylated hydroxytoluene (BHT, 0.01%) and 1 mM ethylene diamine tetra acetic acid (EDTA). Samples were centrifuged at 2000 rpm for 15 min, and 1 mL of each supernatant was mixed with 1 mL of 0.67% thiobarbituric acid (TBA) solution. The reaction mixture was then incubated in a water bath at 90 °C for 20 min and the absorbance were measured at 532 nm using plate reader. The percentage inhibition of TBARS formation relative to the positive control was calculated by

[(A_control_ − A_sample_)/A_control_ × 100)] (2)

where A_control_ is the absorbance of the control and A_sample_ is the absorbance of the test samples.

### 2.6. Pharmacophore Generation for the Active Compounds

A pharmacophore model for the isolated compounds was generated using a Molecular Operating Environment (MOE) software version MOE 2018.01 (Chemical Computing Group, Cambridge, United Kingdom). A database of the isolated compounds with their IC_50_ values was first generated before the pharmacophore. The energy of 3D conformation of the compounds was minimized by MMF94X force field with features including Aro/Pir (radius, 1.4 Å Hyd, Don and acc. 1.0 Å) and Cat and Ani (radius, 1.0 Å). The query cluster was adjusted to 1.25 and conformation to As-Is.

### 2.7. Cyclic Voltammetry Measurement

Cyclic voltammetry (CV) was performed using the BAS100B electrochemical analyzer (Bioanalytical systems, West Lafayette, IN, USA) a glassy carbon electrode (GCE) as working electrode, Ag/AgCl as a reference electrode, and platinum (Pt) as a counter electrode. The CV was recorded at 50 mV/s within the potential window ranging from −1300 mV to 1300 mV, with a measured concentration of samples in phosphate buffer.

## 3. Results and Discussion

### 3.1. Chemical Characterization of the Isolated Compounds

**Figure 1:**
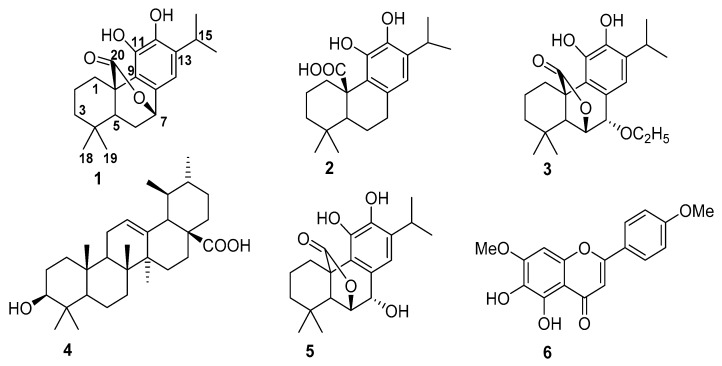
Chemical structures of the isolated compounds.

Chromatographic purification of a methanolic extract of *salvia chameleaegnea* using different techniques including semi Prep-HPLC yielded five pure terpenoids and one flavonoid shown in Figure 1.

#### 3.1.1. Compound 1 (Carnosol)

Compound **1** (0.19 g) was isolated as a white crystal. It was identified as carnosol based on its NMR data (Table 1). ^1^H-NMR showed singlets of two methyl groups at δ_H_ 0.75, and 0.72 (Me-18, 19) and two doublets counted for six protons at 1.06 and 1.05 (Me-16, and 17); septet at 3.36, (H-15), a low field shift proton at 5.26 dd (H-7), a singlet at 6.51 for the aromatic proton H-14. The ^13^C-NMR showed 20 carbons, which confirmed the diterpene skeleton. DEPT-135 and HSQC split those carbons into four methyls (22.4 × 2/C-16, 17; 31.4/C-18 and 19.3/C-19); four methylenes (28.6/C-1; 18.6/C-2; 40.7/C-3; 29.5/C-6); four methines; one of them aromatic (111.6/C-14); and one hydroxylated (78.1/C-7); in addition to the five signals of the 5 aromatic carbons (131.6/C-8; 121.5/C-9; 142.4/C-11; 142.3/C-12; 134.2/C-13) and a carbonyl group (177.5). The heteronuclear multiple bond correlation (HMBC) showed cross peaks (among others) of H-14 with C-15; C-7; C-9; C-11 and/or C-12, H-7/C-5; C-6; C-14; C-9; C-8; C-20; C-12, H-15/C-16,17; C-14; C-13; C-11 and C-12. The above data with 2D NMR experiments {heteronuclear multiple quantum coherence (HSQC), heteronuclear multiple bond correlation (HMBC), and correlation spectroscopy (COSY)} established the structure of carnosol (**1**), which was finally confirmed by comparing the experimental data with literature [14].

#### 3.1.2. Compound 2 (Carnosic Acid)

Compound **2** (0.09g) was isolated as a grey amorphous powder. It was identified as carnosic acid from its NMR data (Table 1). NMR data was similar to that of compound **1** except the absence of the signal at 5.58. The carbon signals showed five methylene (extra one than **1**) and three methines (less one than **1**). The ^13^C signal of the carbonyl carbon was shifted to a lower field (182.1) which indicate free carboxyl group. The above data with comparison of the obtained data with literature confirmed the structure of compound **2** as carnosic acid. Many studies [15] have described the isolation of carnosic acid as its methyl ester derivative due to the fact that, the free carboxyl group makes the chromatographic separation very difficult and also contribute to the instability of the compound during the separation process.

#### 3.1.3. Compound 3 (7-ethoxyrosmanol)

Compound **3** (0.037 g) was isolated as a yellow amorphous powder. It was identified as 7-ethoxyrosmanol based on its NMR data (Table 1). It showed identical signal with that of compound **3** except the chemical shift of C-7 and the presence of ethoxy group in compound **3** (^13^C, 66.4 t; ^1^H, 16.2 q) indicated that 7-ethoxy derivative of **3**. This was confirmed by comparison of the obtained NMR data with those published in literature [16]

#### 3.1.4. Compound 4 (Ursolic Acid)

Compound **4** (0.014 g) was isolated as white powder. It was identified as ursolic acid based on its NMR data, which showed typical triterpene signals with 3 β-OH; carboxylic group (C-28) and C12-13 double bond. The identity of the compound is confirmed by comparing the experimental data with literature [17]. Ursolic acid is a well-known triterpene and widely distributed in plants, the same compound was identified previously from *Leonurus cardiac* [18].

#### 3.1.5. Compound 5 (Rosmanol)

Compound **5** (0.007g) was isolated as brown amorphous solid. The NMR data (Table 1) of **C5** showed a typical abietane diterpene skeleton and similar to compound **1** and **2**. ^1^H NMR showed two doublet signals at 4.49 d (J_6.7_ = 2.3; H-6); 4.66 d (J_6.7_ = 2.3 H-7) 6.80 s (H-14); 3.07 Septet (J_15.6/7_ = 6.8; H-15); 3.11 (m, H-1α in add to four methyl signals, two of them appeared as doublets at 1.19; 1.18 (Me-16; 17; J = 6.8 Hz); and the other two as singlets at 0.88 (Me-18) and 0.87 (Me-19). The ^13^C-NMR and DEPT-135 showed 20 carbons, four of them are methyls at 22.1/C-16 (or C-17); 22.6/C-17 (or C-16); 32.0/C-18 and 20.0/C-19, three methylene signals at 27.3; 19.1 and 38.2 (C-1, C-2 and C-3 respectively); five methines, two of them are oxygenated at 78.2/C-6 and 68.5/C-7 and one aromatic at 120.3/C-14, the other two methines attributed to C-5 (50.8) and C-15/27.3. In addition to seven quaternary carbons, five of them belong to the aromatic ring (128.1, 124.5, 142.9, 141.8, 135.1 (C-8, C-9, C-11–C-13 respectively) and a carbonyl group at 178.9 (C-20); the other two belong to C-10 and C-4 (at 47.2 and 31.3). The chemical shift of C-20 appeared at higher field than compound **2** which indicated the formation of lactone ring also, the H-6 only coupled with H-7 but not with H-5, which indicate the 90° coplanar of H-5 and H-6 and confirm the lactonization at C-6. Finally, the NMR data of rosmanol was identical with those published in literature [14] and established the structure of compound **5** as rosmanol.

#### 3.1.6. Compound 6 (Ladanein)

Compound **6** (0.136 g) was isolated as yellow amorphous powder. The compound showed 15 carbons in addition to two O-Me signals at (δ_H_/δ_C_); 3.87/55.6 and 3.98/56.76. The 15 carbons of the main skeleton indicated Flavonoid type of Apigenin nucleus. The compound showed in NMR signals at 12.57 attributed to 5-OH, and showed cross-peaks in HMBC with C-10 (105.9); C-5 (145.9) and C-6 (129.5). The singlet signal at 6.56 (counted 2H) attributed to H-3 and H-8; it showed HMBC correlations with C-9, C-10, C-7, C-6, [H-8] and C-2; C-4; C-10; C-1 [H-3]. The positions of the two OMe were established by HMBC. The NMR data of ladanein was identical with those published in literature [19] and other 2-D NMR correlations confirm the structure of compound **6** as 6–hydroxy-7,4′-dimethoxyapigenin (ladanein).

### 3.2. Antioxidant and Anti-Tyrosinase Activities of the Isolated Compounds

FRAP and TEAC are based on single electron transfer mechanism while ORAC is based on hydrogen atom transfer [20,21]. The results (Table 2) showed strong activity of carnosol and rosmanol on FRAP, ORAC and TEAC when compared to the reference anti-oxidant epigallocatechingallate (EGCG). Carnosol and rosmanol, and related diterpenes are known to have strong antioxidant activity due to the presence of *o*-dihydroxyl groups in the aromatic ring that serve as hydrogen and/or electron donating agents to the corresponding reactive species leading to the formation of the stable quinone derivatives [22]. It has been reported that the phenolic group at the 11^th^ position (Figure 1) of the molecules contribute significantly to biological activities exhibited by these groups of phytochemicals [23]. It is well known that phenolic compounds are a class of compounds that can create beneficial effects in vivo owing to their antioxidant properties (through radical scavenging) and at the same time show hazardous effects due to their pro-oxidant properties. It is expected that the transfer of electrons or donation of protons to reactive radicals may cause resonance stability of the phenoxyl radical produced [24,25]. However, this is not the case in relation to the experimental results obtained as only few compounds showed strong antioxidant activity. It is assumed that nature of chemical structures of compounds have a major role in the overall bioactivity properties exhibited by each compound. Carnosol and rosmanol, with the highest values for FRAP, TEAC and ORAC share common chemical structure framework which may possibly enhance their bioactivity and mechanism of reactions. Carnosol, carnosic acid and rosmanol, have previously been reported to exhibit remarkable activity [26], which corroborates our findings.

The excessive generation of free radicals in the human body is known to stimulate the activation of tyrosinase enzyme leading to their over-activity and resultant skin hyper-pigmentation [27]. One of the consequences of oxidative stress or damage is the susceptibility of the polyunsaturated lipid membranes to attack as a result of free radical reaction [28]. The results showed that Carnosol and rosmanol, respectively display excellent inhibitory activity against Fe^2+^-induced lipid peroxidation and this is followed by carnosic acid as indicated in Table 3. The significant activity recorded may be due to the presence of vicinal –OH groups in Carnosol, carnosic acid and rosmanol that can chelate with pro-oxidative metals or scavenge peroxyl radical thereby preventing oxidation. The non-reactivity of ursolic acid in lipid peroxidation may be due to lack of a phenolic group, lipophilicity as well as the alkyl group structure saturation in the compound [24]. A similar explanation may hold for the weak reactivity of 7-ethoxyrosmanolpossibly due to lipophilic nature of the compound which could have been enhanced due to the presence of ethoxyl (OC_2_H_5_) group present in the vantage position of the molecule. It is to be noted therefore, that substitution at this position which is common to carnosol, carnosic acid and rosmanol plays very significant role in the bioactivity potency of these compounds.

In the structure-activity relationship (SAR), the occupation of C-7 position is directly related to the activity. The position in the case of carnosic acid is un-substituted and free whereas in rosmanol, the C-7 is occupied by free OH group. Although, the antioxidant activity of carnosol is high, the highly-stress lactone ring may open during the course of chemical reaction leading to extension of conjugation and formation of *p*-quininoidal structure. In 7-ethoxyrosmanol, antioxidant activity is lower compared to carnosol, carnosic acid and rosmanol, possibly due to substitution of C-7 position with ethoxyl group which promotes the hydrophobicity in the compounds. It is generally known that flavonoid compounds as found in ladanein are good antioxidant agents [25]. Although, the anti-tyrosinase activity as demonstrated by the tested compounds is low, nevertheless, the strong antioxidant activity of the compounds serves as important information to establishing the potential of the methanolic extract of *S. chamelaeagnea* (SC) as skin de-pigmenting agent.

### 3.3. Pharmacophore Elucidation for the Antioxidant Activity

**Figure 2:**
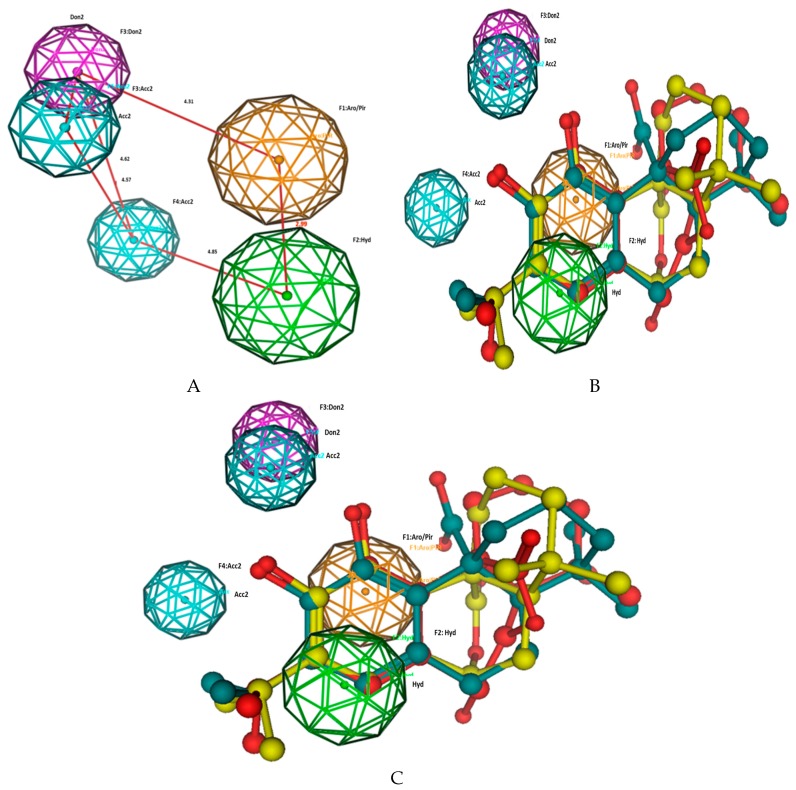
Pharmacophore model and structure requirements of the isolated compounds carnosol, carnosic acid and rosmanol as antioxidant agents. (**A**) Pharmacophiric model of compounds, distances are measured in Å. (**B**) Alignment of the most active compounds carnosol, carnosic acid and rosmanol on the pharmacophore model. (**C**) Superimpositions of the pharmacophore model on rosmanol. Aro/Pir; aromatic, Don; H-bond donner, ACC; H-bond acceptor, Cat and Ani; cationic and anionic.

The generated pharmacophore model gave four structural features shared in the most active compounds named carnosol, carnosic acid and rosmanol (Figure 2). A hydrophobic moiety and aromatic ring are separated by 2.9 Å and placed on the isopropyl and catechol moieties respectively, in the active molecules. In addition, a H-bond donor and acceptor moieties separated by 1.6 Å faced the first OH of the catechol moiety, and another acceptor separated by 4.6 and 4.5 Å from the previously mentioned OH-moiety and faced the second phenolic OH.

### 3.4. Cyclic Voltammetry of Isolated Compounds

**Figure 3:**
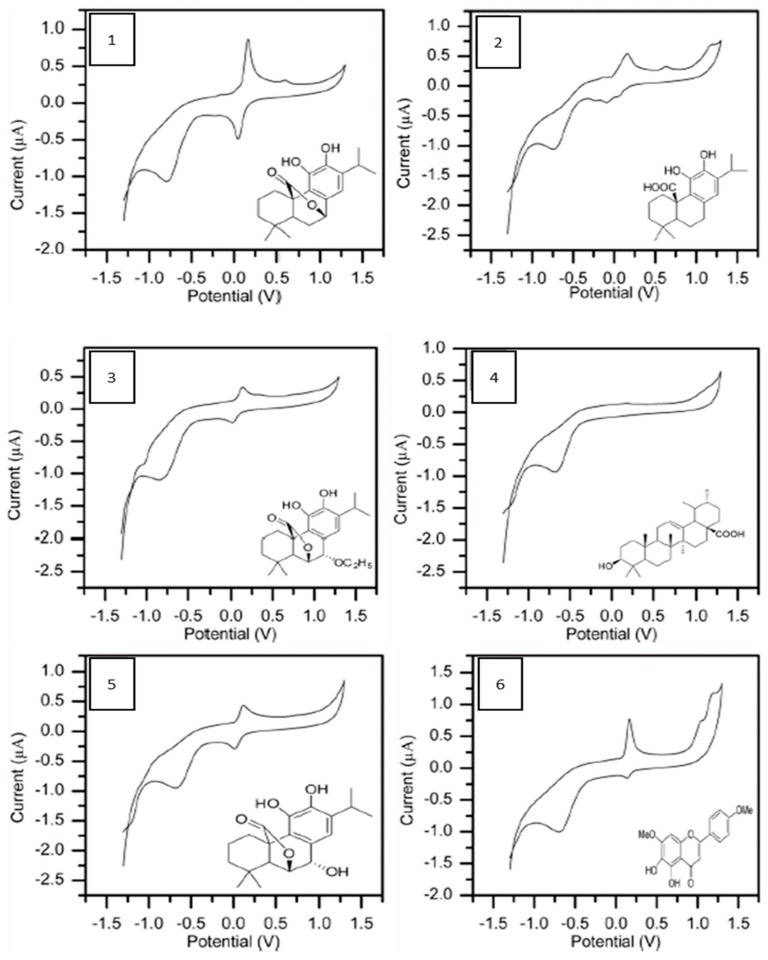
Cyclic voltammograms of isolated compounds. The voltammograms were recorded at 50 mV/s (from −1300 to 1300 mV) for 1 mg/L of each compound in phosphate buffer (pH 6.5).

The cyclic voltammetry (CV) on glassy carbon electrode was used to monitor the oxidation-reduction potentials of all isolated compounds at the broad scan range of−1300 to 1300 mV at 50 mV/s. CV has been reported as one of the techniques used in the characterization of oxidizing and/or reducing the ability of natural phenolics, of which good correlations have been recorded for redox potentials and antioxidant properties. As a result, compounds with strong scavenging capabilities have been reported to be oxidized at relatively low potentials [29,30,31,32,33]. However, the most powerful reducing agents are phenolics with low potential as they can by autoxidation exert pro-oxidant activity [34]. The values of the anodic peak potential (E_pa_) and the anodic peak current (i_pa_), corresponding to the potentials and currents for maximum oxidation of the compounds, are listed in Table 4. As shown in the table, rosmanol when compared to other compounds, exhibits the lowest oxidation potential value (E_pa_ = 0.11 V), indicating that rosmanol has the highest antioxidant power, which is in agreement with ORAC and lipid peroxidation experiments. In this work, all the isolated compounds, except ursolic acid, are electro-active in nature with well-defined oxidation-reduction peaks. The redox activity of these compounds is illustrated in Figure 3. The electro-oxidation may be attributed to the electron transfer and/or hydrogen transfer reactions involving the oxidized product. The difference in the oxidation peak potentials of carnosol, carnosic acid, 7-ethoxyrosmanol and rosmanol is found to be related to the difference in degree of oxidation, which is determined by the number of oxygen species available for the oxidation process; and this is higher for rosmanol than for 7-ethoxyrosmanol. Electron deficiency based on electron-withdrawing effect of a COOH group may also account for the formation of phenoxonium ion as intermediates if positive charge is on a carbon atom. The presence of ortho-hydroxyl group on benzene ring may cause steric effects leading to a shift in potential. The low E_pa_ value of rosmanol (See Table 4) may also be explained in terms of the additional OH- group present in the structure [35,36].

## 4. Conclusions

These studies revealed that *S. chamelaeagnea* methanolic extract is a source of abietane diterpenes which show appreciable antioxidant activity when FRAP, TEAC, ORAC were used as test methods. The electrochemical property of all the compounds isolated and reported was also examined. The effective use of cyclic voltammetry as a reliable analytical method to characterize the reducing ability and electrochemical behavior of phenolic antioxidants in plants has been shown previously. We observed that the measured oxidation potential was closely related to the structures of the investigated compounds. The antioxidant activity of these compounds relates well to the number of OH group in structure, and compounds with two or more electron donating groups have lower anodic peak potentials and higher antioxidant abilities than those with free alcohol or mono-substituted phenols as expressed in ladanein with OCH_3_. Generally, some of the isolated compounds show appreciable antioxidant and lipid peroxidation activity, but a very weak antityrosinase activity was obtained when compared to the crude methanolic extract. We viewed that the low antityrosinase profiles of the tested isolated compounds may not be unconnected with synergism due to the fact that the crude methanolic extract of *S. chamelaeagnea* as the source shows very strong antityrosinase property, however, there is a good correlation between antioxidative activities and oxidation potentials.

## Figures and Tables

**Table 1:** ^1^H- and ^13^C NMR data of 1–3 and 5 in CDCl_3_.

	Compds	Carnosol (1)		Carnosic Acid (2)		Rosmanol (3)		7-Ethoxy-rosmanol (5)	
No.		^1^H	^13^C	^1^H	^13^C	^1^H	^13^C	^1^H	^13^C
1.		2.36 ddd	28.6	1.17	34.4	1.94 ddd	27.3 t	1.96 ddd	27.4 t
		2.71 dddd		3.26 br d		3.11 br d		3.31 br d	
2.		1.51 ddddd	18.6	1.73 br d	20.3	1.46 m	19.1 t	1.64 m	19.0 t
		1.81 ddddd		1.56 m		1.39 m		1.4 m	
3.		1.39 ddd	40.7	1.29 ddd	41.6	1.1 br d	38.2 t	1.09 m	38.0 t
		1.13 ddd		1.44 br d		1.37 m		1.53 m	
4.			34.2		34.5 s		31.3 s		31.4 s
5.		1.6 dd	45.3	1.53 br d	53.9 d	2.15 s	50.8 d	2.26 s	50.9 d
6.		2.06 ddd	29.5	2.37 m	18.9	4.49 d	78.2 d	4.34 d	75.3 d
		1.75 dddd		1.82 m					
7.		5.26 dd	78.1	2.79 m	31.5	4.66 d	68.5 d	4.63 d	75.7 d
				2.79 m					
8.			131.6		129.0		128.1 s		126.6 s
9.			121.5		122.6		124.5 s		124.6 s
10.			48.2		48.7 s		47.2 s		47.0 s
11.			142.3		142.3		142.9 s		142.7 s
12.			142.4		141.2		141.8 s		141.4 s
13.			134.2		133.6		135.1		134.6 s
14.		6.51 s	111.6	6.53 s	119.1 d	6.8 s	120.3	6.77 s	120.8 d
15.		3.07 sept	26.6	3.14 sept	27.0 d	3.07 sept	27.3	3.05 sept	27.2 d
16.		1.06 d	22.4	1.17 d	22.5 q	1.16 d	22.1 q	1.2 d	22.2 q
17.		1.05 d	22.4	1.17 d	22.1 q	1.13 d	22.6 q	1.2 d	22.3 q
18.		0.75 s	31.4	0.97 s	32.6 q	0.95 s	31.4 q	0.99 s	31.3 q
19.		0.72 s	19.3	0.87 s	21.5 q	0.85 s	22.3 q	0.91 s	22.0 q
20.			177.5		182.1		178.9 s	3.83 m	179.1 s
–CH_2_CH_3_								1.31	66.2 t
CH_2_CH_3_									15.8 q

^1^H-NMR: S: singet; d: doublet; sept: septuplet; m: multiplet; br d: broadening doublet; ^13^C NMR: S: secondary; q: quaternary; t: tertiary; dd: two doublets; ddd: three doublets; dddd: four doublets.

**Table 2:** Total antioxidant capacity of *S. chamelaeagnea* constituents.

Sample	FRAP (µM AAE/g)	TEAC (µM TE/g)	ORAC (µMTE/g)
Carnosol	9338.92 ± 1.72	16505.5 ± 0.86	14550.50 ± 3.65
Carnosicacid	4695.98 ± 2.59	5897.5 ± 1.03	10398 ± 1.81
7-ethoxyrosmanol	1113.05 ± 5.6	4618.2 ± 1.11	8247.35 ± 6.83
Ursolic acid	117.26 ± 2.6	NA	2080.19 ± 8.52
Rosmanol	8622.73 ± 1.92	10641.5 ± 0.52	14633.90 ± 3.84
Ladanein	5027.55 ± 4.62	8296.2 ± 1.18	8380.08 ± 4.52
SC	9869.43 ± 7.87	13706.5 ± 0.95	14338.49 ± 5.16
EGCG	4722.51 ± 2.22	10455.1 ± 0.81	14970 ± 5.53

EGCG: Epigallocatechingallate; SC: methanolic extract of *S. chamelaeagnea;* NA: Not active.

**Table 3:** Effects of *S. chamelaeagnea* constituents on inhibition of Fe (II)-induced microsomal lipid peroxidation, anti-tyrosinase activities.

Sample	% Inhibition (μg/mL) Lipid Peroxidation IC_50_	Tyrosinase IC_50_
Carnosol	32.5	455.5
Carnosic acid	38.35	514.9
7-ethoxyrosmanol	>100	465.7
Ursolicacid	>100	485.2
Rosmanol	30.25	>500
Ladanein	>100	554.5
SC	>100	267.4
EGCG	41.50	3.4

Data are given as IC_50_ with tested sample; SC methanolic extract.

**Table 4:** Anodic peak properties of *S. chamelaeagnea* constituents.

Sample	i_pa_/µA	E_pa_/V
Carnosol	0.70	0.16
Carnosic acid	0.33	0.15
7-ethoxyrosmanol	0.18	0.33
Ursolic acid	/	/
rosmanol	0.26	0.11
ladanein	0.60	0.16
SC	0.14	0.15

SC: methanol extract of *S. chamelaeagnea*.

## Supplementary Materials

The following are available online at https://www.mdpi.com/2076-3921/8/4/98/s1.
