# Supplementary material for: Investigation of In-Vitro Antioxidant and Electrochemical Activities of Isolated Compounds from Salvia chamelaeagnea P.J.Bergius Extract
Source: Antioxidants (Basel). 2019 Apr 12;8(4):98. doi: 10.3390/antiox8040098 (PMC6523530; doi:10.3390/antiox8040098)

Investigation of in-vitro antioxidant and electrochemical activities of isolated compounds from *Salvia chamelaeagnea* P.J.Bergius extract

Ninon G.E.R. Etsassala[1], Adewale O. Adeloye[2], Ali El-Halawany[3],Ahmed A. Hussein[2,*] and Emmanuel I. Iwuoha[1]

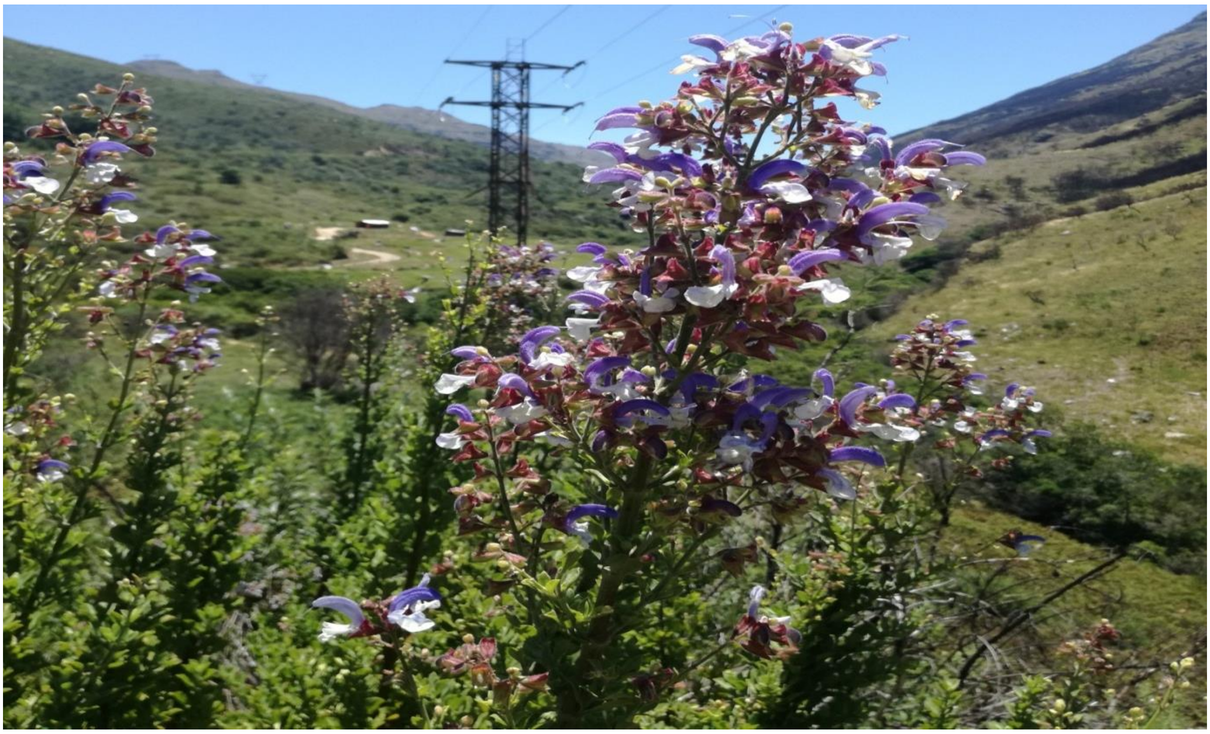

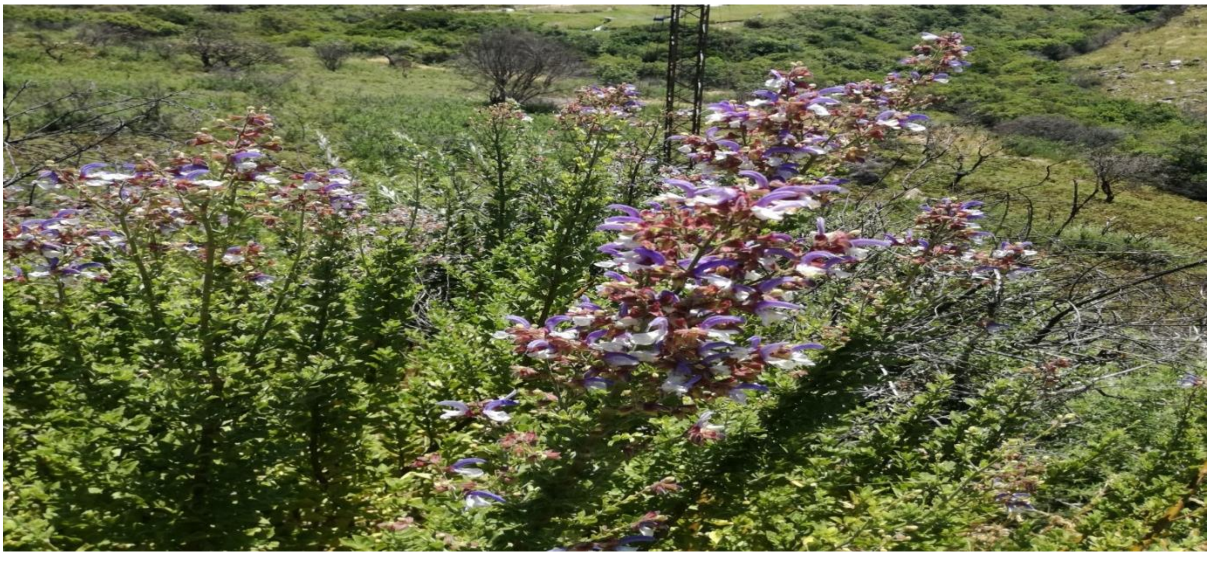

Supplement: Supplementary file 1 [file antioxidants-08-00098-s001.pdf]
